# Biological and Chemical Diversity of Ascidian-Associated Microorganisms

**DOI:** 10.3390/md16100362

**Published:** 2018-10-01

**Authors:** Lei Chen, Jin-Shuang Hu, Jia-Lei Xu, Chang-Lun Shao, Guang-Yu Wang

**Affiliations:** 1 Department of Bioengineering, School of Marine Science and Technology, Harbin Institute of Technology, Weihai 264209, China; m17862700663@163.com (J.-S.H.); Xvjialei2016@126.com (J.-L.X.); 2 Laboratory of Marine Drugs, The Ministry of Education of China, School of Medicine and Pharmacy, Ocean University of China, Qingdao 266003, China; shaochanglun@163.com

**Keywords:** alkaloids, ascidian, bioactivity, diversity, microorganism, polyketides

## Abstract

Ascidians are a class of sessile filter-feeding invertebrates, that provide unique and fertile niches harboring various microorganisms, such as bacteria, actinobacteria, cyanobacteria and fungi. Over 1000 natural products, including alkaloids, cyclic peptides, and polyketides, have been isolated from them, which display diverse properties, such as antibacterial, antifungal, antitumor, and anti-inflammatory activities. Strikingly, direct evidence has confirmed that ~8% of natural products from ascidians are actually produced by symbiotic microorganisms. In this review, we present 150 natural products from microorganisms associated with ascidians that have been reported up to 2017.

## 1. Introduction

Ascidians are the most abundant and diverse class of the sub-phylum Tunicata, and more than 3000 species have been described. They have been found in diverse ecological niches, from shallow water to the deep sea [1]. Thousands of natural products have been isolated from ascidians; these include alkaloids, cyclic peptides, and polyketides [2,3]. Most of these secondary metabolites have diverse bioactivities, such as antibacterial, antifungal, antitumor and anti-inflammatory activities. In addition to the well-known molecules ecteinascidin (ET-743) and didemnin B, several other natural products or their derivatives (e.g., plitidepsin [4], midostaurin [5], lestaurtinib [6], edotecarin [7]) are also in clinical development. However, it has remained unclear whether these bioactive products were produced by ascidians themselves, or by ascidian-associated microorganisms [8,9].

Ascidians harbor rich microbial communities. The development of culture-independent methods has provided comprehensive information about ascidian microbial diversity [3]. In recent years, an increasing number of microorganisms associated with ascidians (including fungi, bacteria, actinobacteria, and cyanobacteria) have been isolated [10]. In this review, we mainly focus on ascidian-associated microorganisms that were isolated by culture-dependent methods.

Microorganisms associated with ascidians represent a potential source of natural products [11]. Many compounds isolated from ascidian-associated microorganisms are extremely potent [12,13,14]. Ecteinascidin 743 (or ET-743, or the trade name Yondelis) was originally isolated from the *Ecteinascidia turbinata* [15]. In 2007, it was approved for the treatment of advanced soft tissue sarcoma by EMEA. In 2011, with the help of metagenomic methods, it was proven that *Candidatus* Endoecteinascidia frumentensis was the actual producer of ET-743 [16]. Didemnin B, originally isolated from the Caribbean ascidian *Trididemnum solidum* [17] was the first marine natural product used in clinical research in the U.S. Recently, researchers have corroborated that didemnin B is produced by the bacterial strains *Tistrella mobilis* and *Tistrella bauzanensis* rather than the ascidians [18,19]. Strong evidence shows that ~8% natural products that were initially thought to originate from ascidians are actually produced by ascidian-associated microorganisms [20].

This review will focus on the biodiversity of ascidian-associated microorganisms, and the chemical structures and bioactive properties of the secondary metabolites isolated from these microorganisms.

## 2. Microorganisms Associated with Ascidians

### 2.1. Geographical Distribution of Microorganisms Associated with Ascidians

**Figure 1:**
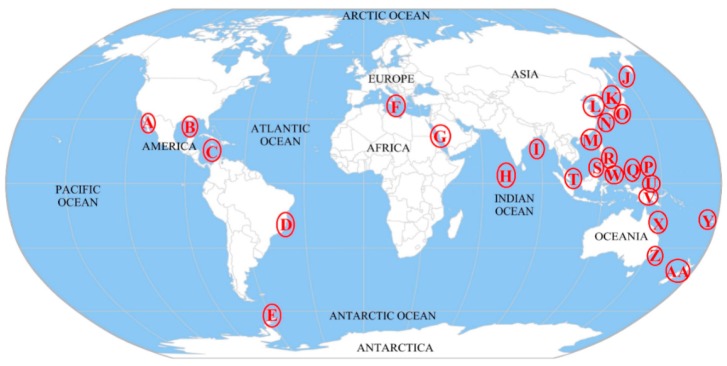
Geographical distribution of ascidian samples used for the research of culturable microorganisms. The red circles represent the sampling sites of research: (A) Baja California; (B) Gulf of Mexico; (C) Caribbean Sea; (D) the coast of Brazil; (E) Antarctic Peninsula; (F) Mediterranean Sea; (G) Red Sea; (H) Maldives; (I) the Bay of Bengal; (J) Kuril Islands; (K) the Sea of Japan; (L) the Yellow Sea; (M) the South Sea; (N) Ryukyu Archipelago; (O) the southeast coast of Japan; (P) Guam; (Q) Palau; (R) Philippine; (S) Celebes Sea; (T) Singapore; (U) Caroline Islands; (V) Papua New Guinea; (W) Micronesian Islands; (X) the Great Barrier Reef; (Y) Fiji; (Z) Tasman Sea; (AA) New Zealand.

Ascidians are widely distributed in oceans around the world. Research on the biological and chemical diversity of microorganisms associated with ascidians has concentrated on the north temperate areas and tropical areas, including Pacific Ocean, Atlantic Ocean, and Indian Ocean. Among these locations, approximately 60% of the sampling sites are located on the southwest coast of the Pacific Ocean (Figure 1). Almost all of these samples are collected from depths shallower than 20 m, and none are from the deep sea.

### 2.2. Diversity of Culturable Microorganisms Associated with Ascidians

Ascidians provide unique ecological niches for a diverse range of microorganisms. The ascidians used for culturable microorganisms belong to 19 genera (*Aplidium*, *Botryllus*, *Ciona*, *Cystodytes*, *Didemnum*, *Diplosoma*, *Ecteinascidia*, *Eudistoma*, *Halocynthia*, *Lissoclinum*, *Oxycorynia*, *Polycitonidae*, *Polyclinum*, *Polycarpa*, *Polysyncraton*, *Pycnoclavella*, *Stomozoa*, *Styela*, and *Trididemnum*) of 10 families (Cionidae, Clavelinidae, Didemnidae, Perophoridae, Polycitoridae, Polyclinidae, Pycnoclavellidae, Pyuridae, Stomozoidae, and Styelidae). The dominant family is Didemnidae. The specific microorganism identity is determined strictly by the precise ascidian species, with which it co-exists [3].

Ascidians can be divided into colonial and solitary classes. Colonial ascidians consist of many small individuals, called zooids, and the whole ascidians were used as the samples for the isolation of microorganisms. Solitary ascidians live as separate individuals with larger bodies, and the corresponding microbial diversity within these diverse ascidian tissues is different [3]. Thus, microorganisms have been isolated from different ascidian tissues, such as the tunic, gonads, gut and pharynx.

To date, diverse microorganisms, such as fungi, bacteria, actinobacteria and cyanobacteria have been isolated from ascidians. Bacteria represent the most abundant class of ascidian-associated microorganisms, and exhibit a high degree of diversity. On the other hand, cyanobacteria have also been widely used to study the symbiosis between microorganisms and their ascidian counterparts.

#### 2.2.1. Bacteria

Ascidians are associated with diverse bacterial populations, and there is species-selective pairing of ascidians and bacteria [21]. For example, bacteria *Acinetobacter* sp. were isolated from the surface of *Stomozoa murrayi* [12], and bacteria *Candidatus* Endoecteinascidia frumentensis was found in symbiosis with *Ecteinascidia turbinate* [16], whereas *Trididemnum solidum* harbours the bacteria *Tistrella mobilis* and *Tistrella bauzanensis* [18,19]. To date, 21 genera belonging to 16 families in four phyla have been cultured from ascidians (Table 1). They are genus *Acinetobacter* belonging to family Moraxellaceae; genus *Agrobacterium* belonging to family Rhizobiaceae; genus *Candidatus* Endoecteinascidia belonging to an unclassified family; genus *Candidatus* Endolissoclinum and *Tistrella* belonging to family Rhodospirillaceae; genus *Endozoicomonas* belonging to family Endozoicomonadaceae; genus *Halomonas* belonging to family Halomonadaceae; genus *Hasllibacter*, *Pseudovibrio*, *Ruegeria*, and *Stappia* belonging to family Rhodobacteraceae; genus *Pseudomonas* belonging to family Pseudomonadaceae; genus *Vibrio* belonging to family Vibrionaceae in the phylum Proteobacteria; genus *Bacillus* and *Paucisalibacillus* belonging to family Bacillaceae; genus *Paenibacillus* belonging to family Paenibacillaceae; genus *Staphylococus* belonging to family Staphylococcaceae; genus *Exiguobacterium* belonging to unclassified family in phylum Firmicutes; genus *Rubritalea* belonging to family Rubritaleaceae in phylum Verrucomicrobia; genus *Labilibacter* belonging to family Marinilabiliaceae and genus *Tenacibaculum* belonging to family Flavobacteriaceae in phylum Bacteroidetes. The dominant phylum of Proteobacteria is represented by 13 genera, which belong to 9 families. Ascidian genus *Didemnum* showed high bacterial diversity, and nearly half of the bacterial genera mentioned in this paper (*Acinetobacter*, *Bacillus*, *Endozoicomonas*, *Exiguobacterium*, *Paenibacillus*, *Paucisalibacillus*, *Pseudomonas*, *Pseudovibrio*, *Ruegeria*, *Staphylococus*, *Stappia* and *Vibrio*) were isolated from them. Surprisingly, culture-dependent and -independent approaches have not often been used to study the symbiosis between bacteria and ascidians, and further work is required in this area [22].

#### 2.2.2. Actinobacteria

The bacterial phylum of Actinobacteria is widely known for the ability to produce bioactive compounds. Marine actinobacteria are widely distributed across different marine ecosystems, such as sediments, water, mangrove, algae, and animals [23,24,25,26]. As is the case with marine invertebrate sponges and corals, ascidians are associated with rich and diverse actinobacteria communities.

A total of 16 genera, belonging to 11 families of phylum Actinobacteria have been isolated from 14 kinds of ascidians (Table 1). *Streptomyces* was the dominant genus and could be found in many ascidians. Fifteen rare actinobacterial genera (*Actinomadura*, *Aeromicrobium*, *Arthrobacter*, *Brevibacterium*, *Curtobacterium*, *Gordonia*, *Kocuria*, *Micrococcus*, *Micromonospora*, *Nocardia*, *Nocardiopsis*, *Saccharopolyspora*, *Salinispora*, *Solwaraspora* and *Verrucosispora*), have also been isolated from various host ascidians.

#### 2.2.3. Cyanobacteria

Cyanobacteria is a phylum of bacteria that produce oxygen during photosynthesis. In 1982, Kott discovered the symbiotic relationship between cyanobacteria and 20 ascidian species. Of these, 17 ascidian species are obligate associates with the symbiotic cyanobacteria genus *Prochloron*, and the other three species (*Trididemnum solidum*, *T. Cyanophorum* and *Didemnum viride*) are associated with the cyanobacteria genus *Synechocystis* [27] (Table 1). Most host ascidians that exhibit symbiosis with the cyanobacteria, *Prochloron*, belong to the Didemnidae family, and are called ‘Didemnid ascidians’. The Didemnidae family also includes some non-symbiotic ascidian species. Genus *Prochloron* is the most representative ascidian symbiont, with *Prochloron didemni* being the sole species in this genus [28]. Cyanobacteria symbionts can both provide nutrients and participate in defence for the ascidian host by means of carbon fixation, nitrogen recycling and metabolite production. In return, the ascidian host can provide some of the nitrogen-containing nutrients that are required for growth of cyanobacteria symbionts, and protect them from ultraviolet radiation [28].

#### 2.2.4. Fungi

The ascidian-associated fungi belong to 25 genera of 19 families in two phyla (Table 1). Most of them belong to the Phylum Ascomycota, which encompasses 22 genera (*Acremonium*, *Alternaria*, *Aspergillus*, *Bionectria*, *Botryosphaeria*, *Botrytis*, *Cladosporium*, *Clonostachys*, *Cochliobolus*, *Epicoccum*, *Fusarium*, *Humicola*, *Meyerozyma*, *Microdiplodia*, *Penicillium*, *Pestalotiopsis*, *Pithomyces*, *Phoma*, *Plectosphaerella*, *Scopulariopsis*, *Talaromyces* and *Trichoderma*). The dominant genus is *Penicillium*, followed by *Aspergillus*, *Cladosporium*, *Talaromyces* and *Trichoderma.* The host ascidians can be classified into 10 genera, of which the dominant genera are *Cystodytes*, *Pycnoclavell*, and *Didemnum*. Host specificity of ascidian-associated fungi is not apparent, and thus the significance of fungi for ascidians and their possible ecological roles remain unclear [29].

## 3. Structure and Bioactivities of Natural Products

To date, 150 natural products have been isolated from ascidian-associated microorganisms. These compounds include polyketides, terpenoids, peptides, and alkaloids. These natural products have diverse properties, such as antimicrobial, antitumor and anti-inflammatory activities.

### 3.1. Polyketides

Polyketides, including macrolides, anthraquinones and polyethers, are derived from the polymerization of acetyl and propionyl groups, and are biosynthesized by three types of polyketide synthases (PKSs). Type I PKSs are multifunctional enzymes, type II PKSs are multienzyme complexes, and type III PKSs are homodimeric enzymes, which are also referred to as ‘chalcone synthase-like PKSs’ [88]. Thirty-seven of the compounds under review here (24.7%) are polyketide-based, and many of them have biological and pharmacological activities.

**Figure 2:**
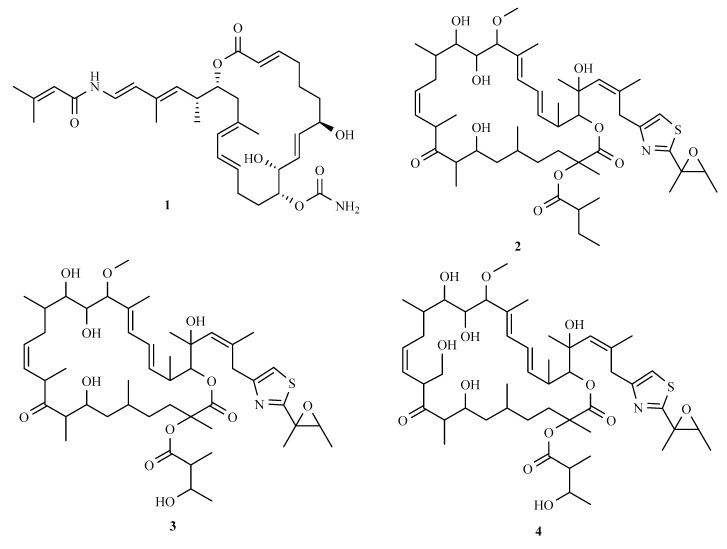

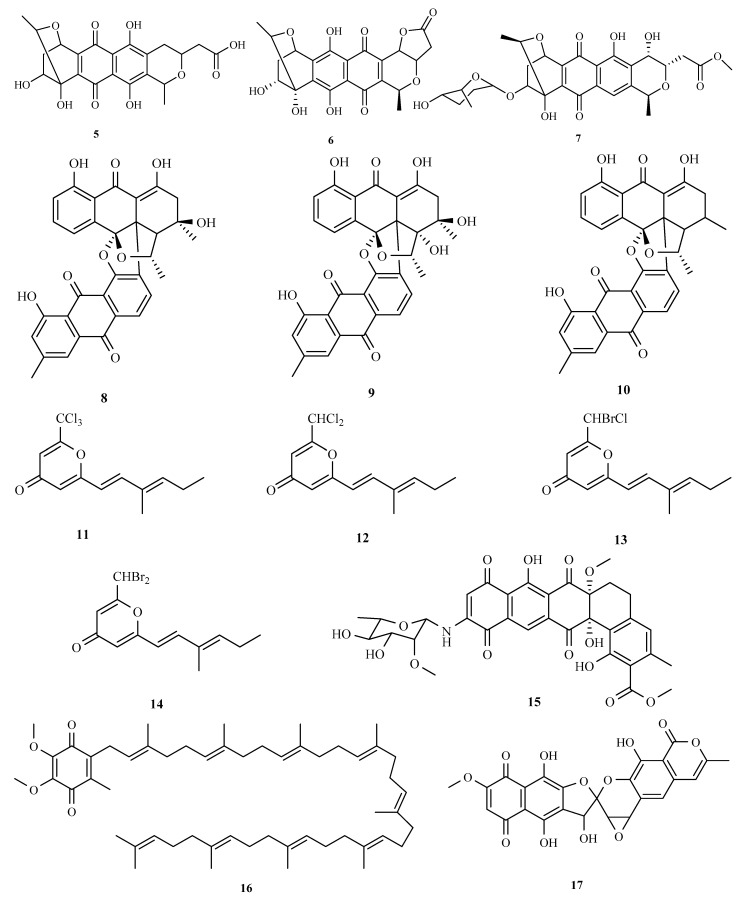

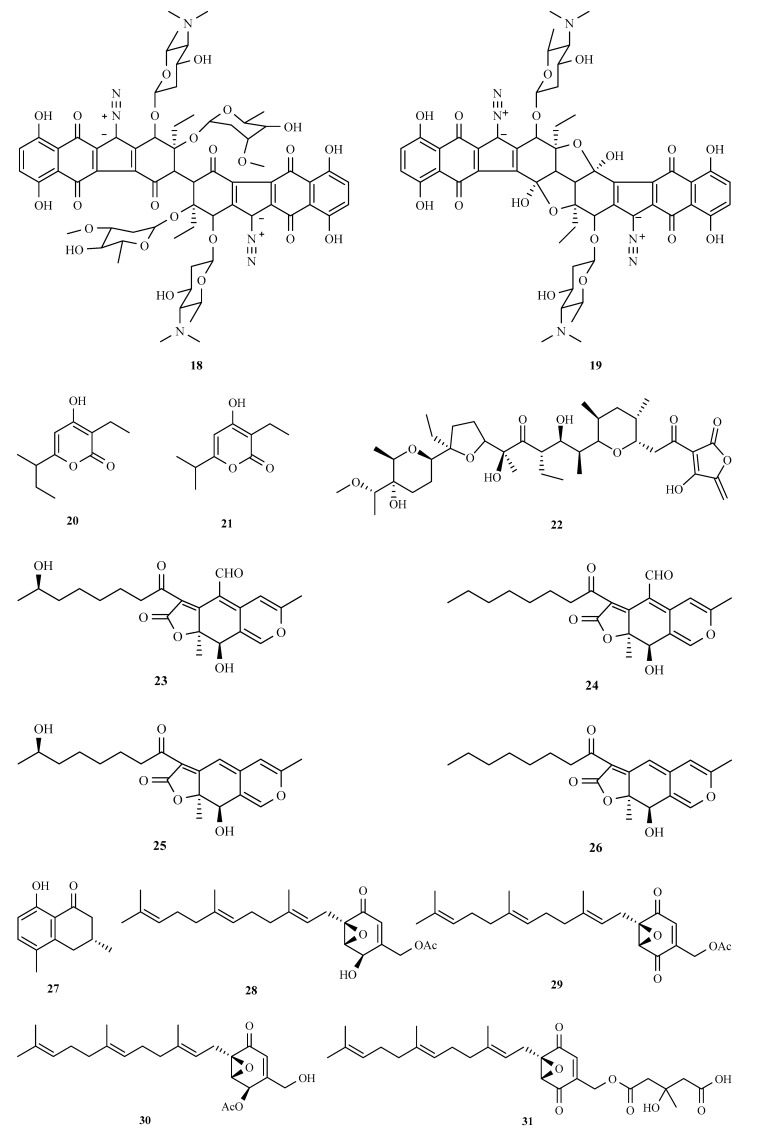

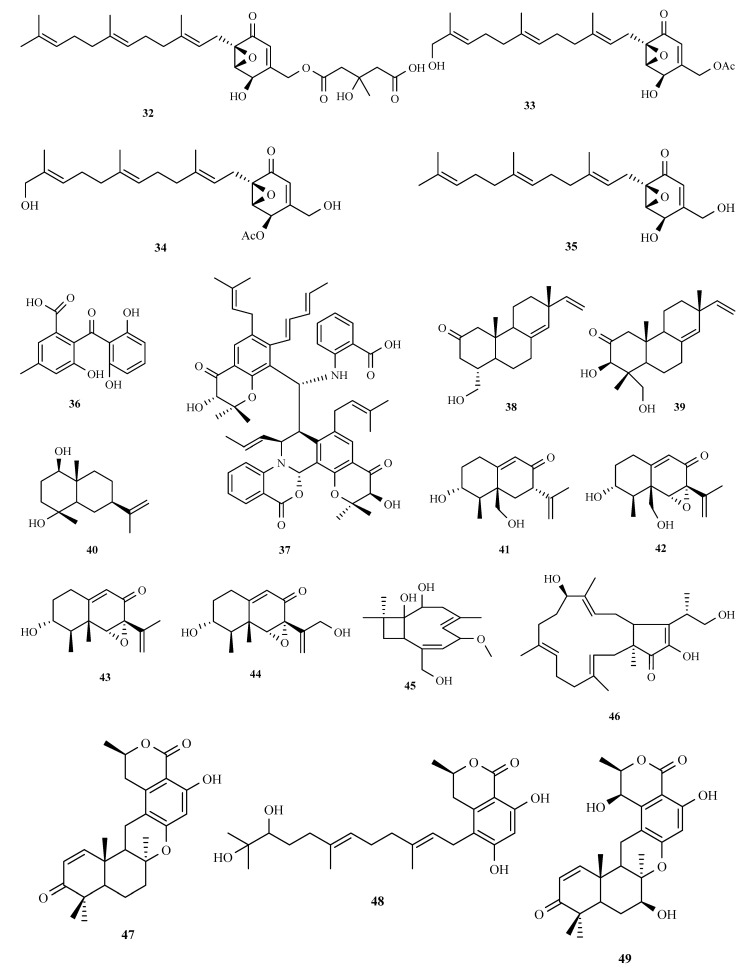

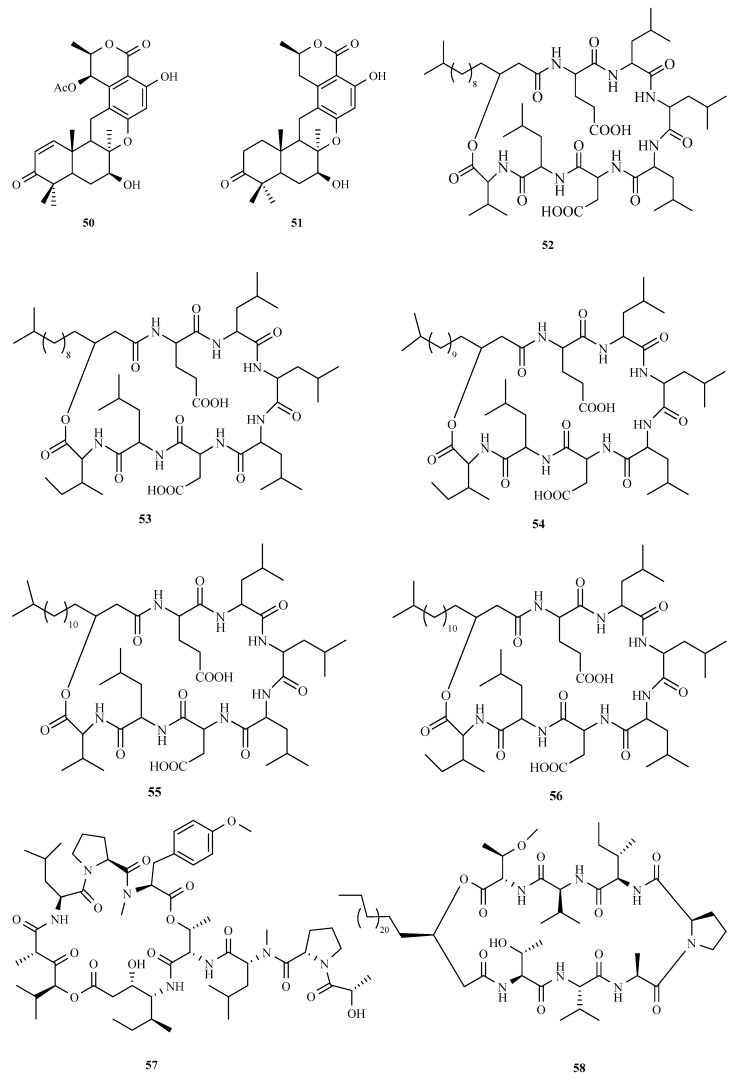

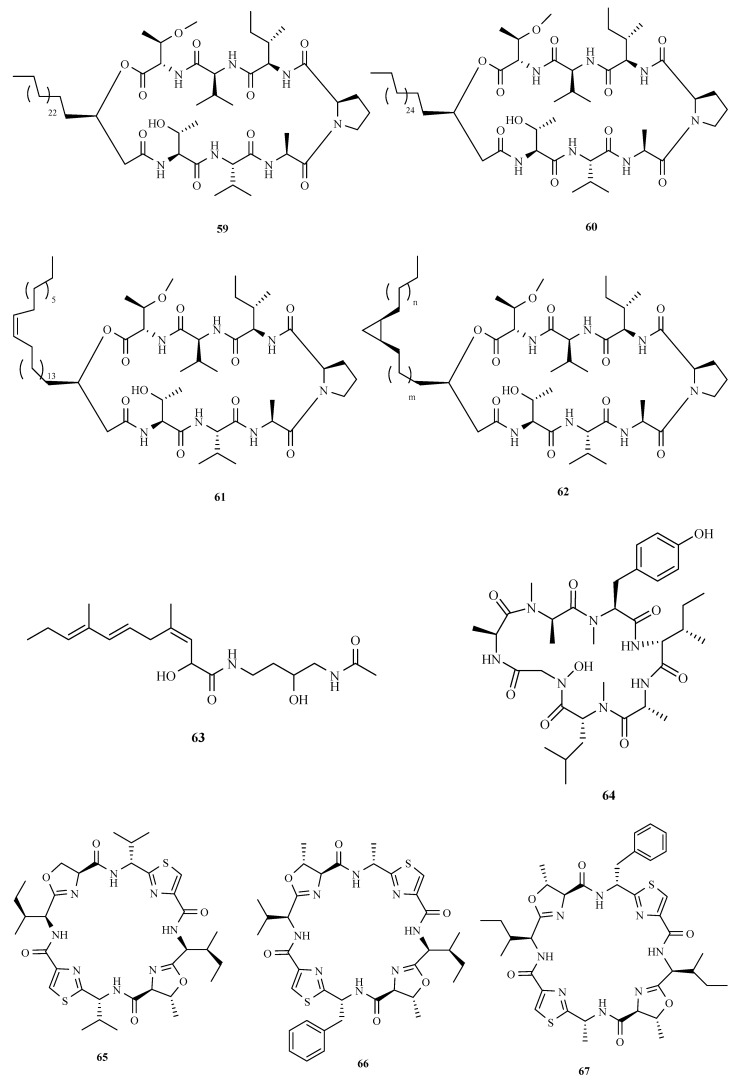

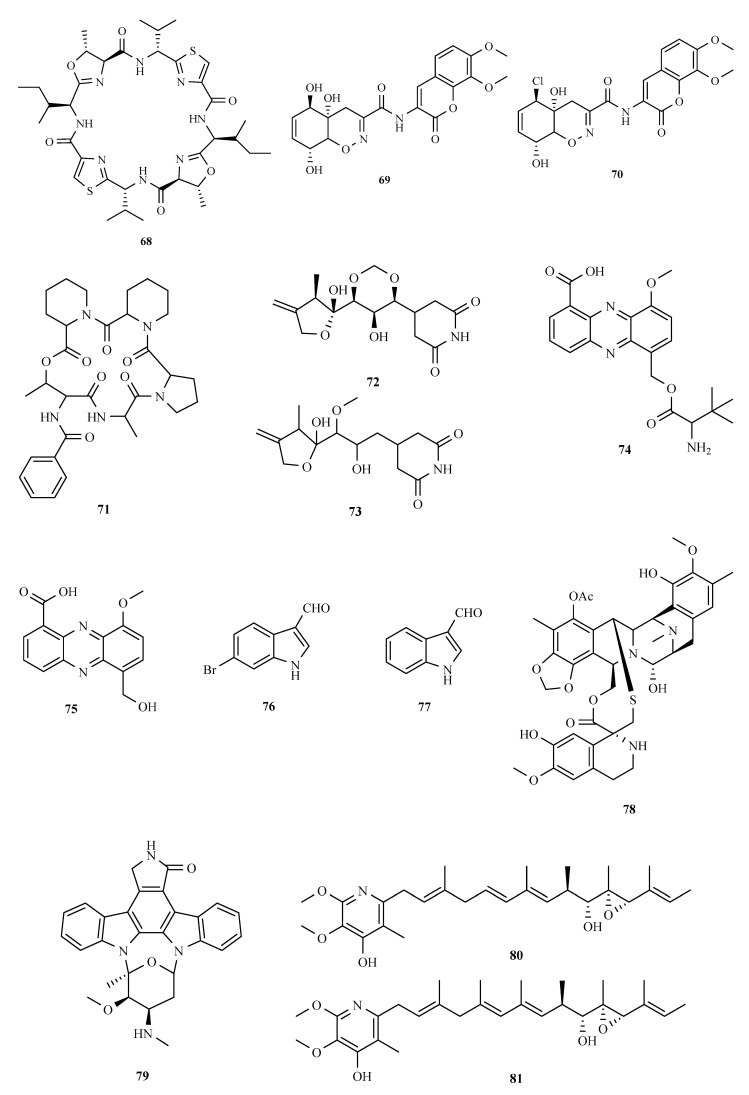

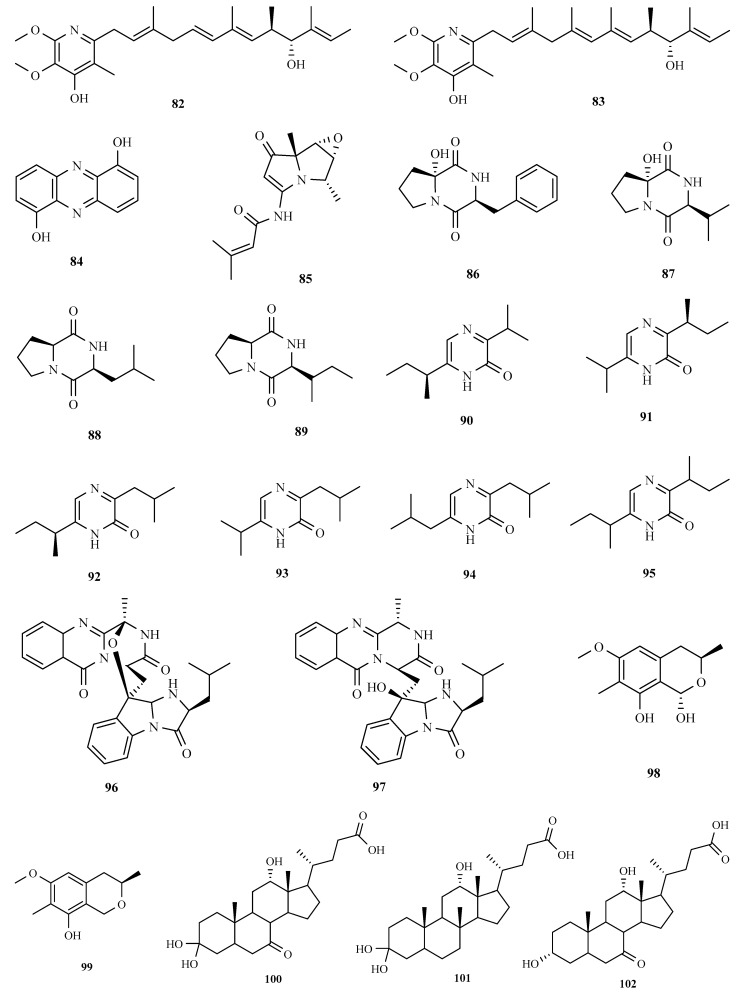

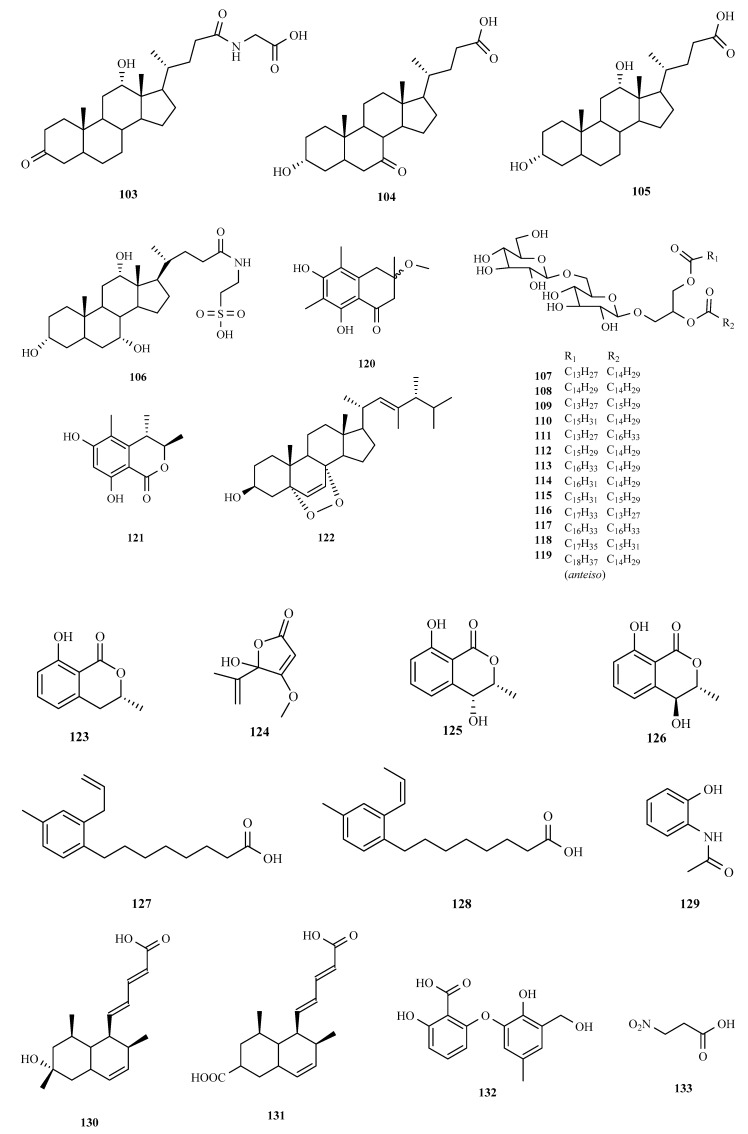

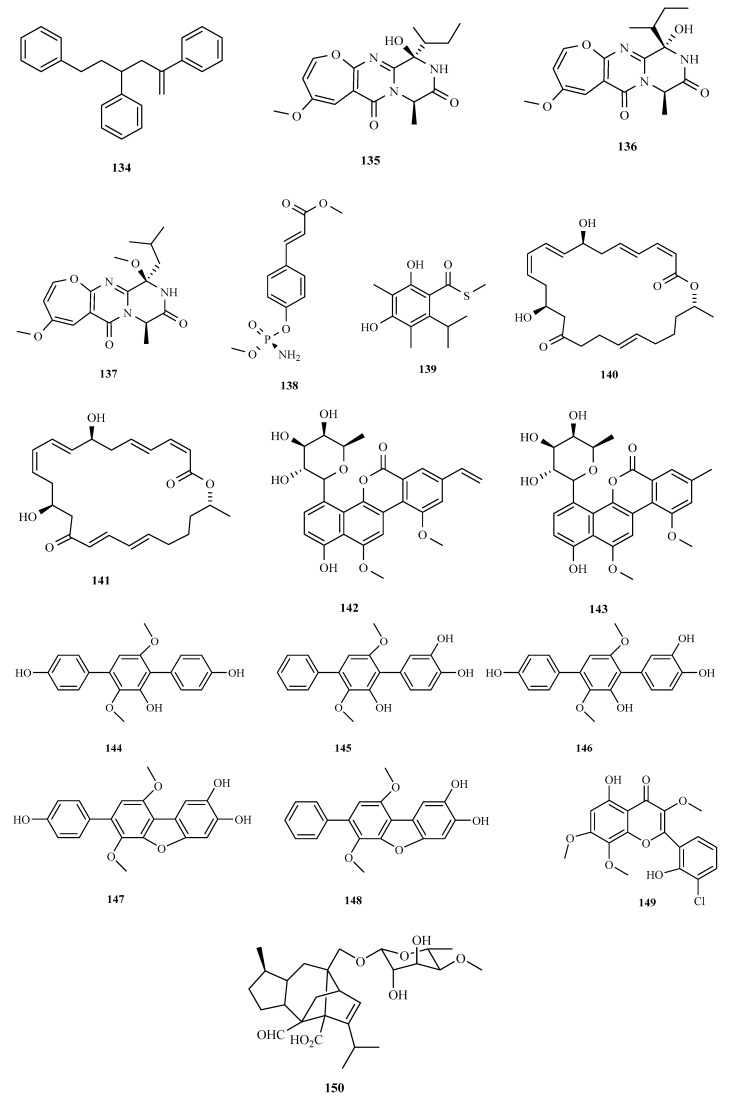
The chemical structures of 150 compounds.

The antimelanoma drug palmerolide A (**1**) (Figure 2), a new enamide-bearing polyketide, was isolated from *Synoicum adareanum*, and was possibly of bacterial origin [89]. It has potent cytotoxicity against melanoma cells (UACC-62, MI14, SK-MEL-5, LOX IMVI), colon cancer cell line HCC-2998 and renal cancer cell line RXF 393. It was also found to be V-ATPase inhibitor [90,91]. Another ascidian, *Lissoclinum patella*, produces patellazoles A–C (**2**–**4**); these natural compounds have strong cytotoxicity against HCT-116 tumour cells [92]. Chemical and biological evidence suggested that the bacterium *Candidatus* Endolissoclinum faulkneri synthesizes patellazoles [34]. Further studies indicate that these products were the foundation of the symbiotic relationship between ascidians and bacteria, and were conserved even during the drive of genome reduction over millions of years [93].

The ascidian-associated bacterium, *Streptomyces* sp. PTY087I2, exhibited enhanced production of three naphthoquinone derivatives, granaticin (**5**), granatomycin D (**6**), and dihydrogranaticin B (**7**), and increased antibacterial activity when co-cultured with the human pathogens *Bacillus subtilis*, methicillin-sensitive *Staphylococcus aureus* (MSSA), methicillin-resistant *Staphylococcus aureus* (MRSA), and *Pseudomonas aeruginosa*) [63]. The isolation of *Streptomyces* sp. #N1-78-1 from *Ecteinascidia turbinata* in Puerto Rico led to the purification of bisanthraquinones 1 and 2 (**8**, **9**), and derivative 3 (**10**), the dehydration product of bisanthraquinone 1. Bisanthraquinones 1 and 2 showed potent antimicrobial activities against MRSA (methicillin-resistant *Staphylococcus aureus*) and VRE (vancomycin-resistant *Enterococcus faecalis*), and these three compounds displayed cytotoxic activity against HCT-116 cells [13]. Two novel chlorinated pyrones, halomadurones A and B (**11**, **12**), and two novel brominated analogues, halomadurones C and D (**13**, **14**) were isolated from *Actinomadura* sp. strain WMMB499 associated with *Ecteinascidia turbinata* in the Florida Keys. Halomadurones C and D showed potent nuclear factor E2-related factor antioxidant response element (Nrf2-ARE) activation, but were toxic at high concentrations [46]. Arenimycin (**15**) was the first report of the benzo[α] naphthacene quinone class of antibiotic isolated from marine actinobacteria *Salinispora arenicola* strain CNR-647, which is associated with *Ecteinascidia turbinate*. Arenimycin exhibited potent antimicrobial activities against drug-resistant *Staphylococci*, some other Gram-positive microorganisms and one *Mycobacterium* strain [14]. Ubiquinone Q9 (**16**), which was determined as 2,3-dimethoxy-5-methyl-6-polyprenyl-1,4-benzoquinone by NMR spectroscopy and mass spectrometry, has been isolated from *Nocardia* sp. strain KMM 3749, a bacterium associated with an unidentified ascidian. This compound inhibited the development of fertilized eggs from the sea urchin *Strongylocentrotus intermedius* and caused haemolysis of mouse erythrocytes [52]. Griseorhodin A (**17**), a member of the rubromycin family, is an inhibitor of human telomerase [94]. The biosynthesis gene cluster for griseorhodin A was isolated from *Streptomyces* sp. JP95, which is associated with *Aplidium lenticulum* collected at Heron Island, Queensland, Australia [60]. In order to find the actual producer of namenamicin, a potent antitumour compound isolated from *Polysyncraton lithostrotum*, a number of actinobacteria were isolated from the inner core of the host ascidian. Among them, the actinobacteria *Salinispora pacifica* (originally proposed to be *Micromonospora lomaivitiensis*), strain LL-37I366 produced two novel lomaiviticin compounds, A and B (**18**, **19**). These natural products, which are members of the angucycline family of aromatic polyketides, contain a distinctive diazotetrahydrobenzo[b]fluorene scaffold also found in the kinamycins [56]. Both compounds were demonstrated to be potent DNA damaging agents by biochemical induction assay (BIA), and have antimicrobial activities against *Staphylococcus aureus* and *Enterococcus faecium*. Lomaiviticin A also showed cytotoxicity against a number of cancer cell lines [55]. The actinobacteria *Streptomyces coelicoflavus* strain HQA809, which is associated with *Styela clava*, produced two natural compounds, germicidin (**20**) and 6-isopropyl group-3-ethyl-4-hydroxy-2-pyrone (**21**). Both of these compounds were lethal to *Artemia salina* [53]. The isolation of *Actinomadura* sp. from *Ecteinascidia turbinata* led to the purification of ecteinamycin (**22**). It showed potent antimicrobial activity against *Clostridium difficile* NAP1/B1/027 [47].

Pitholides A–D (**23**–**26**) and (*R*)-5-methylmellein (**27**) were isolated from the fungus *Pithomyces* sp., which is associated with *Oxycorynia fascicularis* collected from Indo-Pacific. The bioactivities of pitholides A–D (**23**–**26**) were not mentioned, but (*R*)-5-methylmellein (**27**) was lethal in a brine shrimp assay [81]. Yanuthones A–E (**28**–**32**) together with 1-hydroxyyanuthone A (**33**), 1-hydroxyyanuthone C (**34**), and 22-deacetylyanuthone A (**35**) were isolated from the fungus *Aspergillus niger*, which is associated with *Aplidium* sp. The yanuthones showed weak antimicrobial activities against methicillin-resistant *Staphylococcus aureus* and vancomycin-resistant *Enterococcus* sp. The mixed routes for yanuthone biosynthesis imparts structural diversity to this class of compounds [74]. The total synthesis of yanuthones A–C and 22-deacetylyanuthone A has been accomplished following a short regio- and stereocontrolled approach involving the key intermediate, 2-farnesyl-*p*-benzoquinone [95]. A known benzophenone derivative, monodictyphenone (**36**) was isolated from an Indonesian ascidian-associated *Penicillium albobiverticillium* TPU1432, and exhibited moderate inhibitory activities against protein tyrosine phosphatase (PTP) 1B, T cell PTP (TCPTP), CD45 tyrosine phosphatase (CD45), and *vaccinia* H-1-related phosphatase (VHR) [82]. Monodictyphenone (**36**) was previously isolated from the fungal strain *Monodictys putredinis*, which in turn is associated with a marine green alga [96]. A novel filamentous fungus, in the class Eurotiomycetes strain 110162 was isolated from *Lissoclinum patella* collected in Papua New Guinea. A racemic, prenylated polyketide dimer, oxazinin A (**37**), was isolated from this fungus, and was composed of a unique combination of benzoxazine, isoquinoline, and a pyran ring. Oxazinin A showed antimycobacterial activity against *Mycobacterium tuberculosis*, cytotoxic activity against human CEM-TART T-cell leukemia line and modestly antagonized the activity of transient receptor potential (TRP) channels [87].

### 3.2. Terpenoids and Meroterpenoids

The terpenoids are derived from five-carbon isoprene units assembled and modified in thousands of ways, as well as their oxygen-containing derivatives. Terpenes are generally considered to be plant metabolites, although more and more terpenoids are isolated from marine microorganism [97]. The number of terpenoids reported from ascidian-associated microorganisms is very small and most of them are sesquiterpenoids, and these compounds showed diverse bioactivities. The meroterpenoids are natural products of mixed biosynthetic origin, which are partially derived from terpenoids.

Two new terpenoids gifhornenolones A (**38**) and B (**39**), together with a known sesquiterpene compound cyperusol C (**40**) were isolated from actinobacterial strain *Verrucosispora gifhornensis* YM28-088 associated with ascidian. However, only gifhornenolone A was reported to have potent inhibitory activity against the androgen receptor [98].

*Didemnum molle* was the source of fungus *Penicillium* sp. strain SS080624SCf1, and this strain produced two novel sesquiterpenoids JBIR-27 (**41**) and JBIR-28 (**42**), together with two known compounds sporogen-AO1 (**43**) and phomenone (**44**). They showed cytotoxicity against HeLa expect for JBIR-27 [80]. The fungus *Humicola fuscoatra* strain KMM 4629 associated with ascidian produced a new sesquiterpene of the caryophyllene series, fuscoatrol A (**45**), and a known compound 11-epiter-pestacin (**46**). This is the first report of fuscoatrol A, but its acetyl form, pestalotiopsin B, has been isolated from the endophytic fungus associated with the bark and the leaves of *Taxus brevifolia*. These two compounds both showed antimicrobial activities against *Staphylococcus aureus* and *Bacillus subtilis*, and fuscoatrol A also exhibited cytotoxic action on the developing eggs of sea urchin *Strongylocentrotus intermedius* [76].

Two new merosesquiterpenes, verruculides A and B (**47**, **48**), together with chrodrimanins A (**49**), B (**50**) and H (**51**) were all isolated from *Talaromyces verruculosus* (basionym: *Penicillium verruculosum*) strain TPU1311 associated with *Polycarpa aurata*. Compounds **47**, **49** and **51** inhibited the activity of protein tyrosine phosphatase 1B (PTP1B). This was the first study to demonstrate chrodrimanin family as PTP1B inhibitors [84].

### 3.3. Peptides

Peptides isolated from ascidian-associated microorganisms are mainly cyclic. They are nonribosomal peptides (NRPs) synthesized by huge protein complexes called nonribosomal peptide synthetases (NRPSs), and NRPs contain a high proportion of cyclic or branched nonproteogenic amino acids. Most of these cyclopeptides have biological and pharmacological properties, such as antibiotic and antitumor activities [99].

*Bacillus pumilus* strain KMM 1364, which is associated with *Halocynthia aurantium*, produced surfactin-like cyclic depsipeptides 1 (**52**), 2 (**53**), 6 (**54**), 7 (**55**) and 8 (**56**). These peptides, isolated as two C-terminal variants, have a leucine residue in position 4, in contrast to the valine present in the lipopeptide surfactin; the lipophilic parts of the peptide have not been completely characterized [33].

Didemnins A, B, and C, a class of cyclic depsipeptides, were first isolated from the Caribbean ascidian *Trididemnum solidum* in 1981 [17]. These compounds showed significant in vitrocytotoxicity and in vivoantitumor activity [18], and were also active against both DNA and RNA viruses [100]. Didemnin B (**57**) was the first marine compound to enter clinical trials as an antineoplastic agent, and exhibited anticancer activity in phase II clinical trials; however, it ultimately failed as a drug, because of its significant toxicity. Didemnin B now was confirmed to be produced by the marine α-proteobacteria *Tistrella mobilis* [18,19]. Complete genome sequence analysis of the *T. mobilis* strain KA081020-065 discovered the didemnin biosynthetic gene clusters; this lead to the hypothesis that didemnin X and Y precursors may be converted to didemnin B in this organism, which is an unusual post-synthetase activation mechanism [19].

Five new lipopeptide peptidolipins B–F (**58**–**62**) were isolated from the actinobacteria *Nocardia* sp., which is associated with *Trididemnum orbiculatum*. Peptidolipins B and E showed antimicrobial activities against methicillin-resistant *Staphylococcus aureus* (MRSA) and methicillin-sensitive *Staphylococcus aureus* (MSSA) [51]. JBIR-66 (**63**), a new compound isolated from *Saccharopolyspora* sp. strain SS081219 JE-28 (associated with an unidentified ascidian) displayed relatively weak activity against human lymphoblastoid Namalwa cells. The structure of JBIR-66 was identified as (3*Z*,6*E*,8*E*)-*N*-(4-acetamido-3-hydroxybutyl)-2-hydroxy-4,8-dimethylundeca-3,6,8-trienamide on the basis of extensive NMR and MS spectroscopic data [54]. A new compound talarolide A (**64**) was isolated from *Talaromyces* sp. associated with ascidian, and reported to have no antifungal ability [85].

The patellamides are cyclic peptides that exemplify both the unique structural features and potent bioactivities of natural products isolated from ascidians of the Didemnidae family [64]. For example, in 1982 *Lissoclinum patella* was reported to produce cyclic peptide patellamides A–C, all of which contained an unusual fused oxazoline-thiazole unit. Subsequently patellamide D (1993), patellamide E (1992) and patellamide F (1995) were also isolated from *L. patella*. Patellamides A–C have cytotoxic activity against L1210 murine leukaemia cells, whereas patellamide D is a selective resistance-modifying agent [101,102,103,104]. Cyanobacteria of the genus *Prochloron* are obligate symbionts of many didemnid ascidians, and have been identified as the real producers of cyclic peptides of the patellamide class. For example, genetic evidence has shown that *Prochloron didemni* (associated with *Lissoclinum patella*, Republic of Palau) is the source of cytotoxic compounds patellamide A (**65**) and C (**66**) [64,68]. The patellamide biosynthesis gene from *Prochloron* sp. (associated with *Lissoclinum patella*, Great Barrier Reef, Australia) has been expressed in *Escherichia coli*, leading to the production of patellamide D (**67**) and ascidiacyclamide (**68**); both of these molecules are highly cytotoxic [66].

*Trichoderma virens*, a fungus isolated from *Didemnum molle*, produces two modified dipeptide trichodermamides, A (**69**) and B (**70**). The trichodermamides possess a rare cyclic *O*-alkyl-oxime functionality incorporated into a six-membered ring. Trichodermamide B displayed cytotoxicity against HCT-116 and antimicrobial activity against amphoterocin resistant *Candida albicans*, methacillin resistant *Staphylococcus aureus* and vancomycin resistant E*nterococcus faecium* [86]. Depsipeptide JBIR-113 (**71**) was isolated from the fungus *Meyerozyma* sp., which is associated with the ascidian *Ciona intestinalis* in China. This compound was reported to have lethality against brine shrimp *Artemia salina* [77]. This compound was previously isolated from a marine sponge-derived *Penicillium* sp. This compound was previously isolated from a marine sponge-derived *Penicillium* sp. fS36 in Japan, together with JBIR-114 and JBIR-115. These peptides are all of marine origin and contain pipecolic acid, which is very rarely found in natural products [105].

### 3.4. Alkaloids

Alkaloids are structurally diverse compounds generally classified as such, due to the basic character of the molecule, and the presence of at least one nitrogen atom, preferably in a heterocycle [106]. Alkaloids have been isolated from diverse natural organisms, including ascidians and microorganisms.

Sesbanimides A–C were previously isolated from the seeds of the leguminous plant *Sesbania drummondii* [107,108]. Later, sesbanimide A (**72**) was isolated from the bacteria *Agrobacterium* PH-130, which is associated with *Ecteinascidia turbinata* from the Florida peninsula, and sesbanimide C (**73**) was isolated from the bacteria *Agrobacterium* PH-A034C (associated with *Polycitonidae* sp.) along the Turkish coast [31]. Sesbanimide A is one of the most active sesbania alkaloids, with excellent in vitro cytotoxicity against KB cellsand potent in vivo activity against P-388 murine leukaemia [109]. Isolated from bacteria LL-14I352 (associated with an unidentified orange ascidian, Pacific Ocean, Fiji), phenazine compounds LL-14I352 α (or pelagiomicin) (**74**) and β (**75**) have diverse properties, such as antimicrobial activity, and the ability to inhibit DNA, RNA and protein synthesis, DNA-damaging activity; and cytotoxic activity [44]. 6-bromoindole-3-carbaldehyde (**76**) and its debromo analogue indole-3-carbaldehyde (**77**) were isolated from *Acinetobacter* sp. (associated with *Stomozoa murrayi*). Both compounds inhibit the settlement of cyprid larvae from the barnacle, *Balanus amphitrite.* Compound **76** also presented antimicrobial activity against strain SM-S2, strain SM-Z, *Bacillus marinus* and *Vibrio campbellii* [12]. In 1990, the structures of six newly isolated bioactive compounds (ecteinascidins 729, 743, 745, 759A, 759B, and 770) were assigned. The most abundant compound ET-743 showed excellentin vitro cytotoxicity against L1210 leukaemia cells and potentin vivoactivity against P388 murine leukaemia [15]. However, its clinical utility was hampered by inefficient methodologies for isolation of the compound. This led to the development of (semi)-synthetic methods for its large-scale production, which resulted in a novel anticancer agent sold under the brand name Yondelis (Trabectedin) [110]. Trabectedin is the first marine-derived anticancer drug to be approved by the European Union (2007), and is currently approved in more than 70 countries for the treatment of soft tissue sarcoma [111]. In recent years, using metagenomic sequencing of total DNA from the ascidian/microbial consortium, the natural source of ET-743/Yondelis (**78**) was determined to be the bacteria, *Candidatus* Endoecteinascidia frumentensis, which is associated with *Ecteinascidia turbinate* [16].

Indolocarbazole alkaloids, which are staurosporine derivatives, have received great attention as potent inhibitors of phospholipid/Ca^2+^ dependent protein kinase (protein kinase C) [112]. Staurosporine (**79**) was previously isolated from *Eudistoma toealensis* [113]. However, 16S rRNA tag pyrosequencing of the overall bacterial community suggested that two known bacterial producers of staurosporines, *Salinispora* sp. and *Verrucosispora* sp., were abundant in ascidian tissue, suggesting that the staurosporines were of microbial origin [57]. Two new piericidin compounds, C_7_ (**80**) and C_8_ (**81**), together with previously identified piericidins A_1_ (**82**) and A_2_ (**83**), were isolated from the actinobacteria *Streptomyces* sp. YM14-060, which in turn is associated with an unidentified greenish ascidian found in Iwayama Bay, Palau [114]. All of these four compounds showed cytotoxicity against RG-E1A-7 rat glial cells, and also inhibited the growth of Neuro-2a mouse neuroblastoma cells [114]. Compound 1,6-dihydroxyphenazine (**84**) was isolated from *Nocardiopsis dassonvillei* HQA404, which is associated with *Botryllus schlosseri*. This phenazine has antimicrobial activity against *Vibrio anguillarum* and *Vibrio parahaemolyticus*, lethal activity against *Artemia salina*, and enzyme inhibiting activity against Alpha-glucosidase [53]. Bohemamine (**85**) was isolated from *Streptomyces* sp., a bacterial associated with an unidentified ascidian collected from Lyttelton Harbor, New Zealand [115]. Four known diketopiperazine alkaloids, cyclo (6-OH-d-Pro-l-Phe) (**86**), bacillusamide B (**87**), cyclo (l-Pro-l-Leu) (**88**) and cyclo (l-Pro-l-Ile) (**89**), were isolated from actinobacteria *Streptomyces* sp. Did-27, which is associated with the *Didemnum* sp. These compounds exhibited cytotoxic activities against cancer cell lines HCT-116, HepG2 and MCF-7 [62]. Three new 2(1*H*)-pyrazinone derivatives, including (*S*)-6-(sec-butyl)-3-isopropylpyrazin-2(1*H*)-one (**90**), (*S*)-3-(sec-butyl)-6-isopropylpyrazin-2(1*H*)-one (**91**) and (*S*)-6-(sec-butyl)-3-isobutylpyrazin-2(1*H*)-one (**92**), together with the known (1*H*)-pyrazinones analogues deoxymutaaspergillic acid (**93**), 3,6-diisobutyl-2(1*H*)-pyrazinone (**94**) and 3,6-disec-butyl-2(1*H*)-pyrazinone (**95**) were isolated from the actinobacteria *Streptomyces* sp., which is associated with *Didemnum* sp. Expect for compound **91**, all the other compounds presented cytotoxic activities against cancer cell lines HCT-116, HepG2 and MCF-7 [62].

Two new fumiquinazolines H (**96**) and I (**97**) have been isolated from the extracts of fungus *Acremonium* sp., which is associated with *Ecteinascidia turbinate*; they showed weak antimicrobial activity against *Candida albicans* [72]. A new benzopyran compound, 3,7-dimethyl-1,8-dihydroxy-6-methoxyisochroman (**98**) and a known mycotoxin 3,7-dimethyl-8-hydroxy-6-methoxyisochroman (**99**) have been isolated from *Penicillium steckii*, a fungus associated with an unidentified ascidian [78].

### 3.5. Other Types of Compounds Isolated from Ascidian-Associated Microorganisms

Steroids are compounds containing a four-ring structure termed the cyclopentanoperhydrophenanthrene nucleus. Two new cholic acid derivatives named 3,3,12-trihydroxy-7-ketocholanic acid (**100**) and 3,3,12-trihydroxy-7-deoxycholanic acid (**101**) were isolated from *Hasllibacter halocynthiae* strain KME 002^T^, which is associated with *Halocynthia roretzi* [36]. Another four cholic acid derivatives, 3α,12α-dihydroxy-7-ketocholanic acid (**102**), 12-hydroxy-3-keto-glycocholanic acid (**103**), nutriacholic acid (**104**) and deoxycholic acid (**105**) are also produced by *H. halocynthiae* [37]. Cholic acid is predominantly found in the bile of mammals and, as of 2012, has been identified in 11 bacterial strains. Furthermore, strain KME 002^T^ was identified as the first nutriacholic acid-producing bacterium [37]. The marine bacterium *Aeromicrobium halocynthiae* KME 001^T^, which has been isolated from *Halocynthia roretzi* (Gangneung, Korea), produces the natural compound taurocholic acid (**106**) [48].

A mixture of 1(3),2-di-*O*-acyl-3(I)-*O*-β-gentiobiosylglycerols (**107**–**119**) were isolated from *Bacillus pumilus* associated with *Halocynthia aurantium*. The predominant component contains two C15 acyl groups, while the second component contains C15 and C17 fatty acids. Six minor components differ in the number and/or compositions of fatty acids [32].

Two new isocoumarin derivatives, stoloniferols A (**120**) and B (**121**), together with a known sterol, 5α,8α-epidioxy-23-methyl-(22*E*,24*R*)-ergosta-6,22-dien-3β-ol (**122**) were isolated from the fungus *Penicillium stoloniferum* QY2-10, which is associated with an unidentified ascidian. In vitro cytotoxicity assays revealed that **122** was selectively cytotoxic to the P388 cell line when compared to a panel of cancer cells. This is the first report of the cytotoxic activity of **122** [79]. Isocoumarins mellein (**123**), *cis*-4-hydroxymellein (**124**), *trans*-4-hydroxymellein (**125**), and penicillic acid (**126**) were isolated from the fungus *Aspergillus* sp., which is associated with *Eudistoma vannamei*. Only penicillic acid showed cytotoxicity against the tumor cell lines MDA-MB 435 and HCT-8 [75].

Two novel trialkyl-substituted aromatic acids, solwaric acid A (**127**) and solwaric acid B (**128**), were isolated from *Sowaraspora* sp., which is associated with *Trididemnum orbiculatum*; they showed antimicrobial activity against MRSA and MSSA [58]. 2-(acetylamino)-phenol (**129**) was isolated from *Nocardiopsis dassonvillei* strain HQA404, which is associated with *Botryllus schlosseri*., and it showed lethality against brine shrimp *Artemia salina* [53]. Two new carboxylic acids, tanzawic acids E and F (**130**,**131**) were produced by *Penicillium steckii* associated with an unidentified ascidian [78]. A new biphenyl ether derivative 2-hydroxy-6-(2′-hydroxy-3′-hydroxymethyl-5-methylphenoxy)-benzoic acid (**132)** was isolated from Indonesian ascidian-associated *Talaromyces albobiverticillius* (basionym: *Penicillium albobiverticillium*) TPU1432, and exhibited moderate inhibitory activities against protein tyrosine phosphatase (PTP) 1B, T cell PTP (TCPTP), and CD45 tyrosine phosphatase (CD45) [82]. β-nitro-propionic acid (**133**) was isolated from *Humicola fuscoatra*, which is associated with an unidentified ascidian; the compound showed antimicrobial activity against *Staphylococcus aureus*, *Bacillus subtilis*, *Candida albicans* and *Escherichia coli* [76].

The actinobacteria *Solwaraspora* sp., which is associated with *Trididemnum orbiculatum* (Florida Keys) produces 2,4,6-triphenyl-1-hexene (**134**), but this compound has no antimicrobial activity [58]. Three new oxepin-containing natural products, oxepinamides A–C (**135**–**137**) were isolated from *Acremonium* sp., which is associated with the Caribbean ascidian *Ectcinascidia turbinata*; oxepinamide A showed good anti-inflammatory activity in a topical RTX-induced mouse ear oedema assay [72].

*Streptomyces* sp. JP90, which was isolated from *Aplidium lenticulum* (Great Barrier Reef, Australia), produces a new organophosphate (*S*)-cinnamoylphosphoramide (**138**) that displayed inhibitory activity towards BChE [61].

*Streptomyces* sp. isolated from an unidentified ascidian (Lyttelton Harbor, New Zealand) was found to produce a new compound, *S*-methyl-2,4-dihydroxy-6-isopropyl-3,5-dimethylbenzothioate (**139**). This compound is only the fourth natural product reported to contain the *S*-methyl benzothioate group [115]. Macrolactins E (**140**) and F (**141**), together with gilvocarcins M (**142**) and V (**143**) have been isolated from an unidentified ascidian-associated actinobacteria *Saccharopolyspora* sp. SS081219 JE-28 [54].

*Aspergillus candidus* KMM 4676, which was isolated from an unidentified colonial ascidian, produces terphenyllin (**144**), 4″-dehydroxy-3′-hydroxyterphenyllin (**145**), 3′-hydroxyterphenyllin (**146**), candidusin A (**147**), 4″-dehydroxycandidusin A (**148**) and chlorflavonin (**149**). Furthermore, compound **147** and **148** showed cytotoxicity against hormone-sensitive prostate cancer cell line LNCaP [73].

The known diterpene glycoside sordarin (**150**) was produced by *Talaromyces* sp. CMB TU011 isolated from an unidentified ascidian, and it presented antifungal activity [85].

### 3.6. Summary of Natural Products

**Figure 3:**
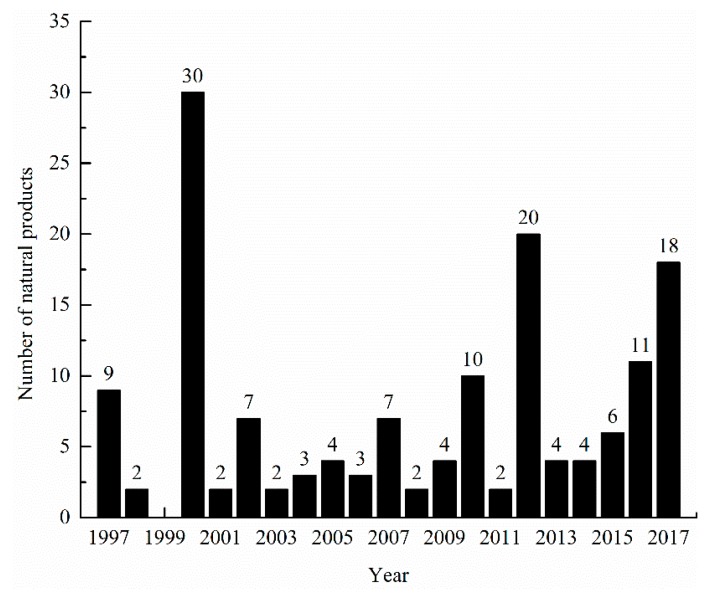
Number of natural products isolated from ascidian-associated microorganisms up to 2017.

**Figure 4:**
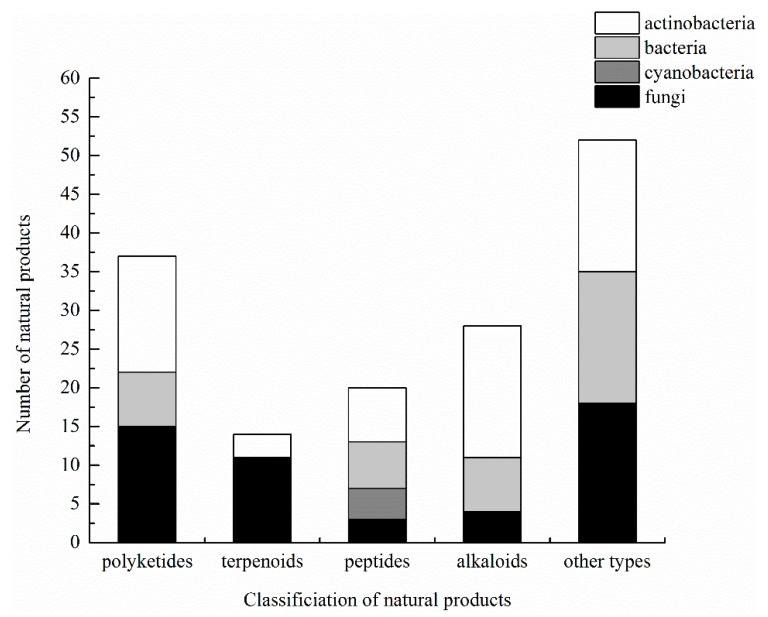
Five groups of natural products isolated from ascidian-associated microorganisms.

**Figure 5:**
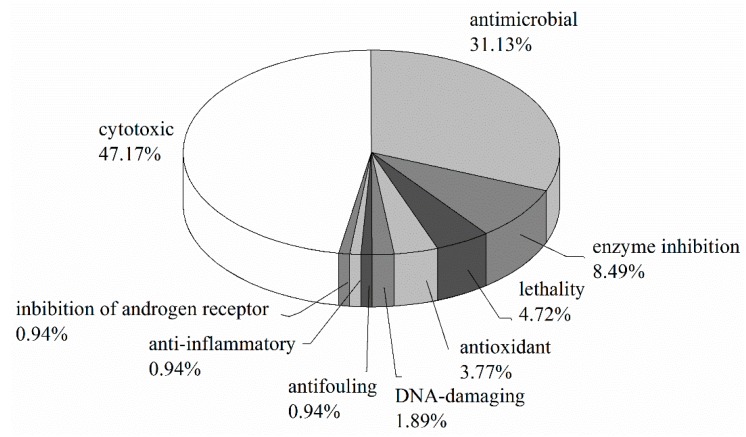
Percentage distribution of bioactivities of natural products from ascidian-associated microorganisms.

150 natural products have been isolated from microorganisms associated with ascidians up to 2017. Natural products originating from ascidian-associated microorganisms is a hot research topic, as evidenced by the surge of publications in this area beginning in the 2000 (Figure 3). Among them, polyketides and alkaloid compounds represent 43.3% of the total number (Figure 4). Most of these compounds have potent bioactivities, and induce in vitro cytotoxicity, or have antimicrobial, anti-inflammatory, antioxidant, and antifouling properties, to name only a few properties (Figure 5). Some compounds have in vivo antitumor activity, and several promising drugs have been used in preclinical evaluation and clinical trials. How far have we progressed in the understanding of the molecular mechanisms of action of these compounds?

**Figure 6:**
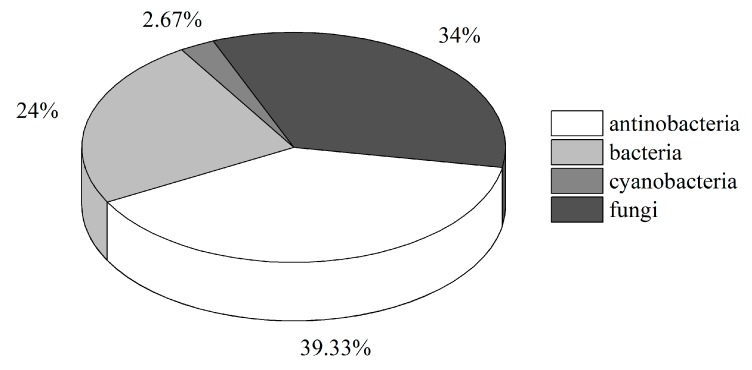
Percentage distribution of natural products isolated from ascidian-associated microorganisms.

Microorganisms are a promising source of bioactive compounds, and the discovery of new strains is vital for new or more active compounds [116]. As discussed in this review, most compounds have been isolated from bacteria, cyanobacteria, actinobacteria, and fungi associated with ascidians (Figure 6). Some compounds were initially considered to be from ascidians, but later confirmed to be produced by ascidian-associated bacteria, such as the well-known ET-743 and Didemnin B; others were isolated directly from the symbiotic microorganisms [32]. Because of the large number of compounds isolated from ascidians, approximately 1080 in 2012 [117], it is important to confirm the true source of more compounds. We think that only the tip of the iceberg has been explored in this regard.

## 4. Conclusions

The marine environment supplies many kinds of habitats that support marine life. It provides an extremely distinct environment for its living organisms. The diverse conditions enable high microbial diversity, and this in turn is associated with biological elaboration of more novel chemical structures [118]. This review has presented 150 natural products produced by ascidian-associated microorganisms. These secondary metabolites belong to polyketides, terpenoids, peptides, alkaloids and other types, and showed a good range of bioactivities. These results indicates the potential of the microorganisms associated with ascidians as sources of bioactive natural products.

In recent years, new approaches to the isolation of microorganisms have been greatly improved. High-throughput cultivation of microorganisms using microcapsules provides an approach to cultivate more biomass. Flow cytometry can then be used to select the microcapsules containing microcolonies. This method can obtain more than 10,000 bacterial and fungal isolates per environmental sample [119]. In 2009, microorganism samples from the coral mucus were encapsulated within agar spheres, encased in a polysulphonic polymeric membrane, and incubated on the mucus surface of coral *Fungia granulosa*. Massive microorganisms obtained shared only 50% similarity (85–96%) with previously identified microorganisms [120]. Alternatively, diffusion growth chambers (DGCs) provide another approach to isolate ‘uncultivable’ microorganisms, as they can be implanted in the tissue of the organism of choice. In 2014, DGCs were first utilized for the cultivation of marine sponge-associated bacteria. Two hundred and fifty-five 16S rRNA gene sequences were obtained, among which 15 sequences were from previously undescribed bacteria [121]. The successful application of new, effective, and efficient approaches in isolating microorganisms will surely contribute to the discovery of novel natural products. However, there are few reports on isolating approaches for ascidian-associated microorganisms. Ongoing studies in our laboratory have been designed to accelerate the isolation of new microorganisms and novel compounds from ascidians.

In closing, we note that with further biotechnological advances, new methods in chemical and biological synthesis will contribute to the discovery of novel and complex drug leads. During the process of finding new compounds, researchers are now sufficiently empowered by such advances that they can think creatively about the drug discovery process. Once the microorganism biosynthetic gene clusters and chemical synthetic routes have been characterized, they can be cloned, artificially modified, and expressed in order to efficiently produce larger amounts of specific compounds, or structurally novel chemical tools [122].

## Figures and Tables

**Table 1:** Microorganism genera associated with ascidians.

Microorganism	Host Ascidian	Geographical Location	Reference
**Bacteria**			
*Acinetobacter* sp.	*Stomozoa murrayi*	AO: Yucatan Peninsula, Mexico	[12]
	*Didemnum ligulum*	AO: São Paulo, Brazil	[30]
*Agrobacterium* sp.	*Ecteinascidia turbinata*	AO: mangroves of the Florida peninsula, US	[31]
	*Polycitonidae* sp.	AO: Turkish coast	[31]
*Bacillus pumilus*	*Halocynthia aurantium*	PO: Sea of Japan	[32,33]
*Bacillus* sp.	*Didemnum* sp.	AO: São Paulo, Brazil	[30]
	*Didemnum ligulum*	AO: São Paulo, Brazil	[30]
*Candidatus* Endoecteinascidia frumentensis	*Ecteinascidia turbinate*	AO: Florida Keys	[16]
*Candidatus* Endolissoclinum faulkneri	*Lissoclinum patella*	PO: Papua New Guinea, Solomon Islands and Fiji	[34]
*Endozoicomonas* sp.	*Didemnum* sp.	AO: São Paulo, Brazil	[30]
*Exiguobacterium* sp.	*Didemnum ligulum*	AO: São Paulo, Brazil	[30]
*Halomonas halocynthiae*	*Halocynthia aurantium*	PO: Sea of Japan	[35]
*Hasllibacter halocynthiae*	*Halocynthia roretzi*	PO: the coast of Gangneung, Korea	[36,37]
*Labilibacter aurantiacus*	*Styela clava*	PO: the Yellow Sea, China	[38]
*Paenibacillus* sp.	*Didemnum ligulum*	AO: São Paulo, Brazil	[30]
*Paucisalibacillus* sp.	*Didemnum ligulum*	AO: São Paulo, Brazil	[30]
*Pseudomonas stutzeri*	*Didemnum* sp.	IO: Maldives	[39]
*Pseudomonas xanthomarina*	*Halocynthia aurantium*	PO: Troitsa Bay, Peter the Great Bay, the Sea of Japan, Russia	[39]
*Pseudovibrio* sp.	*Lissoclinum patella*	AO: São Paulo, Brazil	[30]
*Rubritalea halochordaticola*	Unidentified	PO: Himezu Port, Sado Island, Niigata Prefecture, Japan	[40]
*Ruegeria halocynthiae*	*Halocynthia roretzi*	PO: the South Sea, Korea	[41]
*Ruegeria* sp.	*Didemnum* sp.	AO: São Paulo, Brazil	[30]
	*Didemnum ligulum*	AO: São Paulo, Brazil	[30]
*Staphylococus* sp.	*Didemnum ligulum*	AO: São Paulo, Brazil	[30]
*Stappia* sp.	*Didemnum* sp.	AO: São Paulo, Brazil	[30]
	*Didemnum ligulum*	AO: São Paulo, Brazil	[30]
*Tenacibaculum halocynthiae*	*Halocynthia roretzi*	PO: the South Sea, Korea	[42]
*Tistrella mobilis*	*Trididemnum solidum*	PO: Tateyama cove, Chiba, Japan	[18]
		IO: the Red Sea	[19]
*Vibrio* sp.	*Polyclinum glabrum*	IO: Tuticorin coast	[43]
	*Didemnum* sp.	AO: São Paulo, Brazil	[30]
Unidentified	Unidentified	PO: Fiji	[44]
	*Didemnum* sp.	AO: São Paulo, Brazil	[30]
	*Didemnum ligulum*	AO: São Paulo, Brazil	[30]
	*Ciona intestinalis*	Not mentioned	[45]
**Actinobacteria**			
*Actinomadura* sp.	*Ecteinascidia turbinata*	AO: Florida Keys	[46]
	*Ecteinascidia turbinata*	Not mentioned	[47]
*Aeromicrobium halocynthiae*	*Halocynthia roretzi*	PO: the coast of Gangneung, Korea	[48]
*Arthrobacter* sp.	*Didemnum ligulum*	AO: São Paulo, Brazil	[30]
*Brevibacterium* sp.	*Didemnum ligulum*	AO: São Paulo, Brazil	[30]
*Curtobacterium* sp.	*Didemnum* sp.	AO: São Paulo, Brazil	[30]
	*Didemnum ligulum*	AO: São Paulo, Brazil	[30]
*Gordonia didemni*	*Didemnum* sp.	AO: São Paulo, Brazil	[49]
*Gordonia* sp.	*Didemnum* sp.	AO: São Paulo, Brazil	[30]
*Kocuria* sp.	*Didemnum ligulum*	AO: São Paulo, Brazil	[30]
*Micrococcus* sp.	*Didemnum* sp.	AO: São Paulo, Brazil	[30]
	*Didemnum ligulum*	AO: São Paulo, Brazil	[30]
*Micromonospora* spp.	*Eudistoma vannamei*	AO: Taiba Beach northeastern coast of Brazi	[50]
*Nocardia* sp.	*Trididemnum orbiculatum*	AO: Florida Keys	[51]
	Unidentified	PO: Simushir Island, Kuril Islands	[52]
	*Didemnum ligulum*	AO: São Paulo, Brazil	[30]
*Nocardiopsis dassonvillei*	*Botryllus schlosseri*	PO: the Yellow Sea, China	[53]
*Saccharopolyspora* sp.	Unidentified	PO: Tateyama City, Chiba Prefecture, Japan	[54]
*Salinispora arenicola*	*Ecteinascidia turbinata*	AO: Sweetings Cay, Grand Bahama Island	[14]
*Salinispora pacifica*	*Polysyncraton lithostrotum*	Not mentioned	[55,56]
*Salinispora* sp.	*Eudistoma toealensis*	PO: Islands of Chuuk and Pohnpei, Micronesia	[57]
*Solwaraspora* sp.	*Trididemnum orbiculatum*	AO: Florida Keys	[58]
*Streptomyces hyaluromycini*	*Molgula manhattensis*	PO: Tokyo Bay, Japan	[59]
*Streptomyces* sp.	*Aplidium lenticulum*	PO: Heron Island, Queensland, Australia	[60]
	*Aplidium lenticulum*	PO: Great Barrier Reef, Australia	[61]
	*Didemnum* sp.	IO: Obhur, Saudi Arabia	[62]
	*Ecteinascidia turbinata*	AO: La Parguera, Puerto Rico	[13]
	*Styela clava*	PO: the Yellow Sea, China	[53]
	*Styela canopus*	AO: the Bastimentos National Park in Bocas del Toro, Panama	[63]
*Verrucosispora* sp.	*Eudistoma toealensis*	PO: Islands of Chuuk and Pohnpei, Micronesia	[56]
**Cyanobacteria**			
*Prochloron didemni*	*Lissoclinum patella*	PO: Palau	[64]
*Prochloron* sp.	*Didemnum etiolum*	PO: nothren Great Barrier Reef and Philippine	[27,65]
	*Didemnum molle*	PO: Fiji, Philippine, Palau Island, Lizard Island, northern Great Barrier Reef, Guam and Caroline Islands	[27,65]
	*Diplosoma multipapillata*	PO: Fiji	[27,65]
	*Diplosoma similis*	PO: Caroline Islands, Philippine, Palau, Guam, Norhern Great Barrier Reef and Singapore	[65]
	*Diplosoma virens*	PO: Caroline Islands, Philippine, Palau and Norhern Great Barrier Reef	[65]
	*Echinoclinum triangulum*	PO: Philippine	[27,65]
	*Lissoclinum patella*	PO: Davies Reef, Great Barrier Reef, Australia	[66]
	*Lissoclinum patella*	PO: Philippine, Palau and Guam	[27,65]
	*Lissoclinum punctatum*	PO: Palau and Singapore	
	*Lissoclinum voeltzkowi*	PO: Caroline Islands, Philippine, Palau and Guam	[65]
	*Trididemnum clinides*	PO: Philippine and Guam	[27,65]
	*Trididemnum cyclops*	PO: Palau and Caroline Islands	[65]
	*Trididemnum miniatum*	PO: Norhern Great Barrier Reef	[27,65]
	*Trididemnum nubilum*	PO: Philippine, Fiji and Great Barrier Reef	[27,65]
	*Trididemnum paraclinides*	PO: Palau	[27,65]
	*Trididemnum paracyclops*	PO: Palau, Philippine and Guam	[65]
	*Trididemnum strigosum*	PO: Philippine	[27,65]
*Prochloron* spp.	*Diplosoma simile*	PO: Crawl Key; Isla Cristobal	[67]
	*Lissoclinum patella*	PO: Palau; Palau New Guinea	[68]
	*Lissoclinum verrilli*	PO: Isla Cristobal	[67]
*Synechocystis didemin*	*Didemnum* spp.	PO: Baja, California, Mexico	[69]
*Synechocystis* sp.	*Didemnum viride*	PO: Philippine and Palau	[27,70]
	*Trididemnum cyanophorum*	PO: Panama and Guadaloupe	[27,70]
	*Trididemnum solidum*	AO: Galeta, Panama	[27]
Unidentified	*Trididemnum clinides*	PO: Okinawajima Island, Ryukyu, Archipelago, Japan	[71]
**Fungi**			
*Acremonium* sp.	*Ecteinascidia turbinata*	AO: Bahamas	[72]
*Alternaria* sp.	*Cystodytes dellechiajei*	AO: Mediterranean Sea	[29]
	*Didemnum* sp.	AO: São Paulo, Brazil	[30]
*Aspergillus candidus*	Unidentified	Not mentioned	[73]
*Aspergillus fumigatus*	*Pycnoclavella communis*	AO: Mediterranean Sea	[29]
*Aspergillus niger*	*Aplidium* sp.	PO: Caesar’s Rock in Benga, Fiji	[74]
*Aspergillus* sp.	*Cystodytes dellechiajei*	AO: Mediterranean Sea	[29]
	*Didemnum* sp.	AO: São Paulo, Brazil	[30]
	*Eudistoma vannamei*	AO: Northeast Brazil	[75]
*Bionectria* sp.	*Didemnum* sp.	AO: São Paulo, Brazil	[30]
	*Pycnoclavella communis*	AO: Mediterranean Sea	[29]
*Botryosphaeria* sp.	*Didemnum* sp.	AO: São Paulo, Brazil	[30]
*Botrytis cinerea*	*Cystodytes dellechiajei*	AO: Mediterranean Sea	[29]
*Cladosporium* sp.	*Cystodytes dellechiajei*	AO: Mediterranean Sea	[29]
	*Didemnum fulgens*	AO: Mediterranean Sea	[29]
	*Didemnum* sp.	AO: São Paulo, Brazil	[30]
	*Pycnoclavella communis*	AO: Mediterranean Sea	[29]
*Clonostachys* sp.	*Didemnum fulgens*	AO: Mediterranean Sea	[29]
*Cochliobolus* sp.	*Didemnum* sp.	AO: São Paulo, Brazil	[30]
*Cunninghamella* sp.	*Didemnum* sp.	AO: São Paulo, Brazil	[30]
*Epicoccum nigrum*	*Cystodytes dellechiajei*	AO: Mediterranean Sea	[29]
*Fusarium* sp.	*Cystodytes dellechiajei*	AO: Mediterranean Sea	[29]
	*Didemnum* sp.	AO: São Paulo, Brazil	[30]
*Humicola fuscoatra*	Unidentified	PO: Shikotan island, the Kuril isles	[76]
*Meyerozyma* sp.	*Ciona intestinalis*	PO: the Yellow Sea, China	[77]
*Microdiplodia* sp.	*Didemnum fulgens*	AO: L’Escala, Spain ‘La Depuradora’, Mediterranean Sea	[29]
*Mucor* sp.	*Didemnum* sp.	AO: São Paulo, Brazil	[30]
*Penicillium brevicompactum*	*Cystodytes dellechiajei*	AO: Mediterranean Sea	[29]
	*Didemnum fulgens*	AO: Mediterranean Sea	[29]
*Penicillium rubens*	*Didemnum fulgens*	AO: Mediterranean Sea	[29]
*Penicillium steckii*	Unidentified	AO: Mochima Bay, Mochima National Park and Paria Bay, Irapa, Venezuela	[78]
*Penicillium stoloniferum*	Unidentified	PO: Jiaozhou Bay, Qingdao, China	[79]
*Penicillium* sp.	*Cystodytes dellechiajei*	AO: Mediterranean Sea	[29]
	*Didemnum fulgens*	AO: Mediterranean Sea	[29]
	*Didemnum molle*	PO: Ishigaki Island, Okinawa Prefecture, Japan	[80]
	*Didemnum* sp.	AO: São Paulo, Brazil	[30]
	*Pycnoclavella communis*	AO: Mediterranean Sea	[29]
*Pestalotiopsis* sp.	*Didemnum* sp.	AO: São Paulo, Brazil	[30]
*Phoma* sp.	*Cystodytes dellechiajei*	AO: Mediterranean Sea	[29]
	*Pycnoclavella communis*	AO: Mediterranean Sea	[29]
	*Didemnum* sp.	AO: São Paulo, Brazil	[30]
*Pithomyces* sp.	*Oxycorynia fascicularis*	IO and PO	[81]
*Plectosphaerella* sp.	*Cystodytes dellechiajei*	AO: Mediterranean Sea	[29]
*Rhizopus* sp.	*Didemnum* sp.	AO: São Paulo, Brazil	[30]
*Scopulariopsis* sp.	*Didemnum fulgens*	AO: Mediterranean Sea	[29]
*Talaromyces albobiverticillius (*basionym: *Penicillium albobiverticillium*)	Unidentified	PO: Manado, Indonesia	[82,83]
*Talaromyces verruculosus (*basionym: *Penicillium verruculosum*)	*Polycarpa aurata*	PO: Manado, Indonesia	[83,84]
*Talaromyces* sp.	*Pycnoclavella communis*	AO: Mediterranean Sea	[29]
	Unidentified	PO: Tweed Heads, NSW, Australia	[85]
*Trichoderma harzianum*	*Pycnoclavella communis*	AO: Mediterranean Sea	[29]
*Trichoderma virens*	*Didemnum molle*	PO: Madang, Papua New Guinea	[86]
*Trichoderma* sp.	*Didemnum fulgens*	AO: Mediterranean Sea	[29]
	*Didemnum* sp.	AO: São Paulo, Brazil	[30]
Unidentified	*Didemnum* sp.	AO: São Paulo, Brazil	[30]
Unidentified (A fungus in the class Eurotiomycetes)	*Lissoclinum patella*	PO: Papua New Guinea	[87]

Symbols of world principal oceanic areas: AO, Atlantic Ocean; ArO, Arctic Ocean; IO, Indian Ocean; PO, Pacific Ocean; SO, Southern Ocean (Antarctic Ocean).
